# Deciphering the Antioxidant and Neuropharmacological Effects of Methanolic Extract of *Artocarpus chama* Leaves and Its n‐Hexane and Ethyl Acetate Fractions: GC–MS Analysis, Experimental, and In Silico Investigations

**DOI:** 10.1002/fsn3.71227

**Published:** 2025-11-19

**Authors:** Md. Lokman, Md. Liakot Ali, Qurratul Ain Sadia, Saad Ahmed Sami, Md. Ashraful Alam, Nawreen Monir Proma, Mohammad Rashedul Islam, Mohammed Kamrul Hossain

**Affiliations:** ^1^ Department of Pharmacy, Faculty of Biological Sciences University of Chittagong Chittagong Bangladesh; ^2^ Department of Pharmacy Jashore University of Science and Technology Jashore Bangladesh

**Keywords:** antidepressant, antioxidant, anxiolytic, *Artocarpus chama*, GC–MS, molecular docking, sedative

## Abstract

*Artocarpus chama*, a widely known fruit‐bearing plant in Asia and locally referred to as Chapalish in Bangladesh, has been traditionally used for various medicinal purposes. However, its neuropharmacological potential remains largely unexplored in current scientific literature. This research aims to investigate the in vitro antioxidant and in vivo neuropharmacological properties of the methanolic extract of *A. chama* leaves (MEAC) along with its n‐hexane (NHAC) and ethyl acetate (EAAC) fractions. The DPPH assay revealed strong antioxidant activity, with MEAC displaying an IC₅₀ of 24.30 ± 1.04 μg/mL, while NHAC and EAAC showed IC₅₀ values of 45.18 ± 2.4 μg/mL and 30.46 ± 1.15 μg/mL, respectively. In in vivo models, MEAC, NHAC, and EAAC exhibited significant anxiolytic effects in the elevated plus maze and hole‐board test at both doses (200 and 400 mg/kg). Notable sedative effects were observed in the open field test at 200 and 400 mg/kg doses, but only in the hole cross test at the 400 mg/kg dose. Additionally, all three extracts demonstrated significant antidepressant activity in behavioral despair tests at all investigated doses. GC–MS analysis identified a total of 40 phytoconstituents, several of which exhibited notable binding affinities to key drug target proteins involved in oxidative stress, anxiety, sedation, and depression, as shown through molecular docking studies. Furthermore, ADMET predictions supported their drug‐likeness and favorable pharmacokinetic and toxicity profiles. These findings suggest that *A. chama* leaves may serve as a promising source of bioactive compounds with neuropharmacological potential. However, further extensive studies are warranted.

## Graphical abstract

The methanolic extract and solvent fractions (n‐hexane and ethyl acetate) of *Artocarpus chama* leaves demonstrated significant in vitro antioxidant and in vivo anxiolytic, sedative, and antidepressant activities. GC–MS profiling revealed forty compounds, several showing strong docking affinities with targets related to oxidative stress and neurobehavioral disorders, indicating that *A. chama* leaves could be a promising source of neuroprotective phytochemicals.

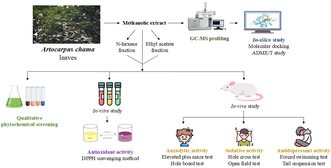

## 1 Introduction

Neuropsychiatric disorders, including depression, anxiety, and insomnia, have become prominent contributors to mood disturbances and behavioral changes in recent years. These disorders impact over 9% of the global population and disrupt daily functioning (Taylor et al. 2005). Often occurring together, anxiety, depression, and insomnia present challenges in accurate diagnosis and effective treatment. Whether individually or in combination, these conditions contribute significantly to disability‐adjusted life years and greatly diminish the quality of life in affected individuals (Diniz et al. 2019).

Anxiety is the most common neuropsychiatric condition, affecting individuals across all age groups. Globally, it impacts over 264 million people, with an estimated 33.7% of the population likely to experience an anxiety disorder at some point in their lives (Avilés‐Montes et al. 2023). Similarly, depressive disorders are becoming increasingly widespread. As per WHO estimates, around 4.4% of the world's population suffers from depression. Depression is projected to become one of the leading causes of disability and illness, second only to heart disease. Additionally, anxiety and depression frequently coexist, often being diagnosed together in the same individuals (Ghosh et al. 2021). Insomnia is a condition characterized by persistent sleep disturbances and is now recognized as a widespread and chronically disabling issue. It places a substantial health and economic burden on both individuals and society. This condition includes difficulties in initiating sleep, maintaining uninterrupted sleep, experiencing fragmented sleep, or waking up earlier than desired (Akram et al. 2019). Insomnia or the symptoms of chronic insomnia affect nearly one‐third of adults globally (Bhaskar et al. 2016). Common pharmacological options for treating anxiety and insomnia include benzodiazepines, barbiturates, and azapirones, while depression is typically managed with medications like atypical antidepressants, tricyclic antidepressants, and monoamine oxidase inhibitors. However, the use of central nervous system (CNS)‐active drugs is often limited by side effects such as addiction, memory loss, sedation, cognitive issues, delayed therapeutic response, sexual dysfunction, and anticholinergic symptoms, which negatively impact treatment compliance (Ali et al. 2025; Dahchour 2022; Diniz et al. 2019). Therefore, the search for new therapeutic agents with effective neuropharmacological properties and fewer adverse effects continues.

The *Artocarpus* genus (family Moraceae) consists of evergreen trees found throughout the tropical regions of Asia. While they are mainly recognized for producing large, edible fruits, several species also hold medicinal significance and have been traditionally utilized in folk medicine practices in countries such as Indonesia, Thailand, Taiwan, and Sri Lanka (Hossen et al. 2025). Traditionally, *Artocarpus* species have been used to address numerous health issues, including diarrhea, diabetes, malaria, tapeworms, wound healing, syphilis, and respiratory problems. They are also believed to have expectorant effects and are used to manage conditions, such as anemia, asthma, and dermatitis (Jagtap and Bapat 2010). *Artocarpus chama*, locally called Chapalish in Bangladesh, is found in various Asian nations, including China, Malaysia, Bhutan, India, Laos, Myanmar, and Thailand. In Bangladesh, it thrives in the mountainous forest areas, especially in the Chattogram Hill Tracts (Sohag et al. 2025). Studies have demonstrated several pharmacological activities of *A. chama* leaves, including antioxidant, cytotoxic, antibacterial, anthelmintic, analgesic, antidiarrheal, and antihyperglycemic effects (Hossen et al. 2025; Sohag et al. 2025). Nonetheless, its role in managing neuropsychiatric conditions remains unexplored in current scientific literature. As far as we are aware, no prior research has studied the neuropsychiatric effects of extracts from *A. chama* leaves or any of their individual phytoconstituents.

Therefore, the present research investigates the in vitro antioxidant activity and in vivo neuropharmacological properties of the methanolic extract of *A. chama* leaves (MEAC) and its n‐hexane (NHAC) and ethyl acetate (EAAC) fractions, using Swiss albino mice in various behavioral models. Furthermore, molecular docking and ADMET analyses were carried out to understand the potential action mechanisms and pharmacokinetic attributes of the plant's bioactive constituents.

## 2 Methods

### 2.1 Chemicals and Reagents

All analytical procedures were conducted using high‐grade reagents obtained from reputable suppliers. The solvents used—methanol, n‐hexane, and ethyl acetate—as well as 2,2‐Diphenyl‐1‐picrylhydrazyl (DPPH), were sourced from Sigma–Aldrich Ltd. (Saint Louis, USA). The standard drugs used in the biological studies, ascorbic acid, diazepam, and fluoxetine, were purchased from Square Pharmaceuticals Ltd., Bangladesh.

### 2.2 Collection of Plant Materials

Leaves of *A. chama* were collected in September 2023 from the University of Chittagong, located in Chittagong district, Bangladesh. The plant species was taxonomically verified by Professor Dr. Shaikh Bokhtear Uddin from the Department of Botany, University of Chittagong, and documented under the voucher number CU/Pharm 1080.

### 2.3 Extract Preparation and Fractionation

The collected leaves were thoroughly cleaned, sun‐dried, and ground into fine powder. Approximately 750 g of the powdered material was soaked in 4 L of methanol at room temperature for 15 days. After the extraction period, the mixture was filtered first through cotton and then through Whatman No. 1 filter paper. The methanolic filtrate was concentrated using a rotary evaporator under reduced pressure and temperature. Subsequently, 5 g of the concentrated methanolic extract underwent fractionation using a modified Kupchan partitioning technique (Kupchan et al. 1975). The resulting fractions were concentrated with a rotary evaporator, yielding 1.7 g of n‐hexane‐soluble fraction (NHAC) and 1.3 g of ethyl acetate‐soluble fraction (EAAC). All residues were stored in a refrigerator until further use.

### 2.4 Qualitative Phytochemical Screening

Qualitative phytochemical screening of MEAC, NHAC, and EAAC was performed using the protocol described by previous studies (Lokman et al. 2025; Shaikh and Patil 2020) to detect the presence of various classes of phytochemicals.

### 2.5 Gas Chromatography–Mass Spectrometry (GC–MS) Analysis

GC–MS analysis was performed to investigate the phytochemical profile of MEAC using a Clarus 690 gas chromatograph (PerkinElmer, CA, USA), equipped with an HP‐5MS column (30 m length × 0.25 mm internal diameter × 0.25 μm film thickness) and coupled with a Clarus SQ 8 C mass spectrometer (PerkinElmer, CA, USA). A 1 μL sample was injected in splitless mode, and high‐purity helium (99.99%) was used as the carrier gas at a constant flow rate of 1 mL/min for a total run time of 60 min. The analysis was conducted in electron ionization (EI) mode with an ionization energy of 70 eV. While the injector temperature remained steady at 280°C, the oven temperature was initially set at 60°C (held for 0 min), then increased at a rate of 4°C per minute until reaching 240°C, which was maintained for 15 min (Ali, Meem, et al. 2024). The chemical constituents were identified by comparing their mass spectra to those available in the NIST library database.

### 2.6 In Vitro Antioxidant Assay

To assess the antioxidant capacity of MEAC, NHAC, and EAAC, the DPPH radical scavenging method was employed according to the procedure described by Nyalo et al. (Ochieng Nyalo et al. 2022).

### 2.7 Experimental Animals and Ethical Approval

Male Swiss albino mice (5–6 weeks old, weighing 20–30 g) were sourced from the Animal House of the Department of Pharmacy, University of Chittagong. Prior to experimentation, they were kept under standardized conditions (25°C ± 2°C temperature, 55%–65% humidity, and a 12‐h light/dark cycle) with free access to standard food and water. The study was approved by the Animal Ethical Review Board, Faculty of Biological Sciences, University of Chittagong, Bangladesh (approval no. AERB‐FBS‐CU‐20231023).

### 2.8 Experimental Design

Mice were randomly divided into eight groups (*n* = 5 per group) to assess pharmacological activities. Group one served as the control and received only the vehicle (1% Tween‐80). Group two was treated with a reference drug specific to each test. The remaining six groups were given MEAC, NHAC, and EAAC at two different doses (200 and 400 mg/kg). Diazepam was used as the standard anxiolytic and sedative agent, while fluoxetine served as the standard antidepressant.

### 2.9 Acute Toxicity Investigations

An acute oral toxicity test was conducted as per protocol by Sany et al. (2025). Five animals per group were given a single dose of MEAC, NHAC, and EAAC (1000, 1500, or 2000 mg/kg). After administration, food was restricted for 3–4 h. Animals were observed closely for 30 min, then periodically for 24 h, and followed for 3 days to detect any toxic signs. Parameters such as skin, fur, eyes, mucous membranes, breathing, circulation, and nervous system activity were also assessed.

### 2.10 In Vivo Anxiolytic Evaluation

#### 2.10.1 Elevated Plus‐Maze (EPM) Test

The EPM apparatus included two open arms and two enclosed arms (50 × 10 × 40 cm^3^), constructed from wood and elevated 40 cm above the ground. Mice were divided and treated as described in the Section 2.8. After 60 min, the mice were placed in the maze's center, facing the closed arms. The time spent in each arm and the number of entries into each arm were recorded over 5 min, and the test was conducted in a soundproof room (Ali, Meem, et al. 2024).

#### 2.10.2 Hole Board Test (HBT)

The HBT apparatus consisted of a wooden chamber (40 × 40 × 25 cm) with 16 evenly spaced holes (3 cm in diameter), elevated 25 cm above the floor. Mice were treated according to the Section 2.8. One hour posttreatment, each mouse was observed for 5 min, during which both the frequency and duration of head pokes were recorded (Ali, Meem, et al. 2024).

### 2.11 In Vivo Sedative Evaluation

#### 2.11.1 Hole Cross Test (HCT)

The hole cross apparatus was a stainless‐steel cage measuring 30 cm by 20 cm by 14 cm. In the center of the cage, a 7.5 cm high divider with a 3 cm diameter hole was installed. Mice were assigned to treatment groups according to the Section 2.8. After 1 h of treatment, the mice were placed in the center of the hole‐cross apparatus. The number of times each mouse crossed the hole was recorded over 3 min at 0, 30, 60, 90, and 120 min following treatment (Ali, Meem, et al. 2024).

#### 2.11.2 Open Field Test (OFT)

The OFT setup consisted of a white square box (60 cm × 60 cm × 60 cm), divided into 25 equal squares (5 cm × 5 cm each). Mice were placed into treatment groups based on the Section 2.8. After 60 min of treatment, the mice were positioned at the center of the box and observed for 3 min. The number of squares crossed during this period was recorded, and further observations were made at 30, 60, 90, and 120 min posttreatment to track the mice's movement (Ali, Meem, et al. 2024).

### 2.12 In Vivo Antidepressant Evaluation

#### 2.12.1 Forced Swimming Test (FST)

A transparent cylindrical container (15 × 25 cm^2^) filled with water (25°C ± 2°C) to a depth of 10 cm was used for this test. Mice were divided and treated as per the Section 2.8. After 60 min, each mouse was placed in the FST apparatus. The test lasted 6 min, with the first 2 min allowing the mice to acclimate and the remaining 4 min recording the time spent immobile (Ali, Meem, et al. 2024).

#### 2.12.2 Tail Suspension Test (TST)

This test followed the protocol described in the Section 2.8 with minor modifications. Sixty minutes after treatment, mice were suspended 50 cm above the ground by adhesive tape placed 1 cm from the ends of their tails. The total time spent immobile over 6 min was recorded, with immobility defined as quiet or still hanging (Ali, Meem, et al. 2024).

### 2.13 In Silico Investigation

#### 2.13.1 Molecular Docking

##### 2.13.1.1 Ligand Preparation

GC–MS analysis of MEAC identified 40 small compounds, which were obtained from PubChem in 3D SDF format. Compounds available only in 2D SDF format were converted to 3D using Open Babel (O'Boyle et al. 2011). The ligands were minimized and saved in .pdbqt format using AutoDockTools (version 1.5.6) (Xue et al. 2022).

##### 2.13.1.2 Protein Preparation

Human erythrocyte catalase (PDB ID: 1DGH), monoamine oxidase A (PDB ID: 2Z5X), the bromodomain of human BRD4 (PDB ID: 3U5J), and serotonin transporter (PDB ID: 5I6X) were retrieved from the RCSB Protein Data Bank for evaluating antioxidant, anxiolytic, sedative, and antidepressant effects. The proteins were processed using Discovery Studio 2020 (Studio 2008) by removing water molecules and other complex structures, followed by energy minimization with Swiss‐PDB Viewer (Guex and Peitsch 1997). The structures in PDB format were then converted to PDBQT format using AutoDockTools (Xue et al. 2022), and the final structures were stored in PDBQT format.

##### 2.13.1.3 Molecular Docking Analysis

Molecular docking in drug design investigates how ligands interact with macromolecular targets, assessing binding free energy and molecular recognition (Azme et al. 2025). This study focuses on explaining the interaction mechanisms of compounds with drug targets related to antioxidant and neuropsychiatric conditions. Docking of selected proteins with plant ligands was performed using PyRx AutoDock Vina (Dallakyan and Olson 2015; Eberhardt et al. 2021), employing a semiflexible system with rigid proteins and flexible ligands. AutoDock defined the grid box around the active site, while BIOVIA Discovery Studio Visualizer was used to visualize both 2D and 3D docking interactions.

#### 2.13.2 ADMET Investigations

To assess the pharmacokinetic (ADME) and toxicological properties of the compounds, two widely used online servers, SwissADME (Daina et al. 2017) and PKCSM (Pires et al. 2015), were utilized. Adherence to Lipinski's Rule of Five (Ro5) was also considered to evaluate the favorable drug‐like properties of the compounds.

### 2.14 Statistical Analysis

The data were expressed as the mean ± standard error of the mean (SEM). Statistical analysis was performed using one‐way ANOVA, followed by Dunnett's t‐test. Differences from the control group were considered statistically significant at *p* < 0.001, *p* < 0.01, and *p* < 0.05. All statistical analyses were conducted using GraphPad Prism software (version 5.2).

## 3 Results

### 3.1 Qualitative Phytochemical Screening

The preliminary phytochemical evaluation of MEAC, NHAC, and EAAC confirmed the presence of alkaloids, glycosides, steroids, phenols, flavonoids, tannins, saponins, and resins (Table 1).

**TABLE 1:** Qualitative phytochemical screening of methanolic extracts of leaves of *Artocarpus chama* as well as its *n*‐hexane and ethyl acetate fractions.

Sl. No.	Phytochemicals	Test	MEAC	NHAC	EAAC
1	Alkaloids	(a) Mayer's test	+++	+	++
		(b) Wagner's test	++	−	
		(c) Hager's test	−	+	+
		(d) Dragendroff's test	−	−	+
2	Carbohydrates	(a) Molisch's test	−	−	+++
		(b) Benedict's test	+	−	++
		(c) Fehling's test	−	+	+
3	Glycosides	(a) Legal's test	+	−	−
		(b) Modified Borntrager's test	+	−	+
4	Saponins	(a) Froth test	+	+	+
		(b) Foam test	+	−	++
5	Phytosterols	(a) Salkowski's test	−	+	−
		(b) Libermann‐Burchard's test	−	+	−
6	Phenols	(a) Ferric Chloride test	+++	+	+
		(b) Bromine water test	++	++	+
7	Tannins	(a) Ferric Chloride test	++	−	+
		(b) Gelatin Test	+	−	−
8	Flavonoids	(a) Alkaline reagent test	++	+++	+
		(b) Lead acetate test	++	++	+
9	Proteins and amino acids	(a) Xanthoproteic test	−	−	+
		(b) Ninhydrin test	−	−	−
10	Terpenes	(a) Salkowski's test	+++	++	+
		(b) Libermann‐Burchard's test	++	+	−
11	Fixed oils and fats	(a) Saponification test	+	+++	−
		(b) Spot test	++	+	+
12	Resins	(a) Acetic anhydride test	+	++	+
		(b) Acetone test	+++	+	−

*Note:* (+) and (−) indicate presence and absence of phytochemicals, respectively.

### 3.2 GC–MS Profiling

**FIGURE 1:**
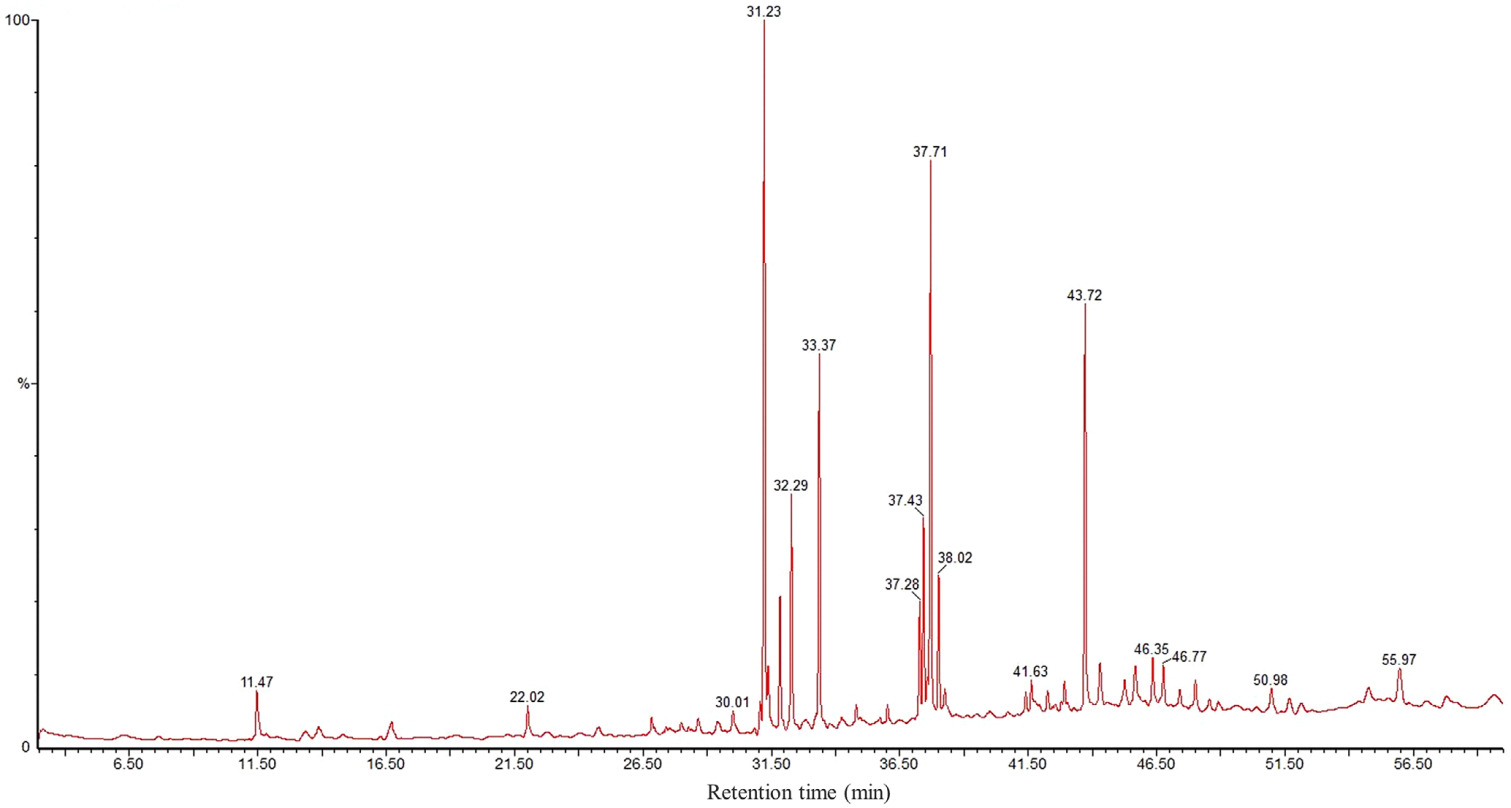
GC–MS chromatogram of methanolic extract of *Artocarpus chama* leaves.

A total of 40 compounds were identified in the MEAC through GC–MS analysis. The chromatogram is presented in Figure 1, and Table 2 outlines the corresponding chemical details of the detected compounds.

**TABLE 2:** Phytochemical composition of methanolic extract of *Artocarpus chama* leaves.

Serial No.	Retention time (RT)	Name of the compounds	Molecular weight	% Area
1	3.04	1H, 3H‐furo[3,4‐c]furan, tetrahydro—	114	0.01
2	7.65	Benzyl alcohol	108	0.22
3	9.41	1‐butanone, 4‐(dimethylamino)‐1‐phenyl—	191	0.18
4	10.23	Octanoic acid, methyl ester	158	0.1
5	11.47	Benzylcarbamate	151	1.8
6	11.83	3,5‐methano‐2,3,4,5 (1H)‐tetrahydrobenzo(b)azepin‐2‐one	173	0.42
7	13.37	Phenylalanine	165	0.54
8	16.29	2‐methoxy‐4‐vinylphenol	150	0.13
9	18.68	Chloroacetic acid, tetradecyl ester	290	0.1
10	19.28	N‐hydroxy‐2‐phenyl‐acetamide	151	0.47
11	24.8	Fumaric acid, butyl tetrahydrofurfuryl ester	256	0.51
12	27.4	4H‐cyclopentacycloocten‐4‐one, decahydro—	166	0.22
13	28	Tetracontane‐1,40‐diol	594	0.53
14	28.28	Tridecanoic acid, 12‐methyl‐, methyl ester	242	0.17
15	28.66	6‐hydroxy‐4,4,7a‐trimethyl‐5,6,7,7a‐tetrahydrobenzofuran‐2 (4H)‐one	196	0.57
16	29.4	Perhydrophenalene, (3a.alpha., 6a.alpha., 9a.alpha., 9b.beta.)—	178	0.57
17	31.23	Neophytadiene	278	12.96
18	31.38	Pentyl dotriacontyl ether	536	1.59
19	31.84	Phytyl tetradecanoate	506	2.58
20	32.29	Phytyl palmitate	534	4.85
21	32.84	Trans, trans‐1,6‐dimethylspiro[4.5]decane	166	0.61
22	33.37	Tetradecanoic acid, 10,13‐dimethyl‐, methyl ester	270	7.43
23	34.24	Tetradecanoic acid	228	0.51
24	35.74	Methyl 11‐methyl‐dodecanoate	228	0.2
25	36.02	2‐hydroxy‐1,8‐naphthyridine	146	0.41
26	37.28	Methyl 10‐trans,12‐cis‐octadecadienoate	294	1.74
27	37.71	Phytol	296	9.53
28	38.02	Heptadecanoic acid, 16‐methyl‐, methyl ester	298	2.39
29	40.7	Docosanoic anhydride	662	0.21
30	41.63	Glycidyl palmitate	312	1.06
31	42.26	Heptacosanoic acid, 25‐methyl‐, methyl ester	438	0.51
32	42.92	9‐octadecenenamide, (z)—	281	0.77
33	43.72	Hexanedioic acid, bis (2‐ethylhexyl) ester	370	7.94
34	45.26	1, 2, 3, 4‐tetrahydro‐3‐(phenylacetamido)quinoline	266	0.96
35	45.68	Hentriacontane	436	1.65
36	46.77	Bis(2‐ethylhexyl) phthalate	390	1
37	48.02	Tetrapentacontane	758	1
38	50.98	Triacontane	422	1.03
39	52.14	Octadecanoic acid, 11‐methyl‐, methyl ester	312	0.52
40	54.76	Octadecane, 2,6,10,14‐tetramethyl—	310	2.46

### 3.3 In Vitro Antioxidant Assay

In the DPPH radical scavenging assay, MEAC demonstrated an IC₅₀ value of 24.30 ± 1.04 μg/mL, while its fractions, NHAC and EAAC, showed IC₅₀ values of 45.18 ± 2.4 and 30.46 ± 1.15 μg/mL, respectively. These results are fairly close to the IC₅₀ of the standard antioxidant, ascorbic acid (19.33 μg/mL), indicating considerable antioxidant activity of the extracts, with MEAC displaying the highest potency among them.

### 3.4 Acute Toxicity Investigations

No adverse effects or deaths occurred with MEAC, NHAC, or EAAC, even at the highest tested dose of 2000 mg/kg. Therefore, a safe lower dose of 200 mg/kg, representing one‐tenth of this maximum dose, and a higher dose of 400 mg/kg, twice that amount, were selected for subsequent experimentation.

### 3.5 In Vivo Anxiolytic Activity

#### 3.5.1 Elevated Plus Maze Method

In the EPM test, significant anxiolytic effects were observed at both doses (200 mg/kg and 400 mg/kg) of MEAC, NHAC, and EAAC, with EAAC showing the most pronounced effect, as indicated by the longest duration spent (86.20 ± 4.02 s) and the highest number of entries (10.20 ± 1.83) in the open arms (Table 3).

**TABLE 3:** Anxiolytic effects of methanolic extract of *Artocarpus chama* leaves and its *n*‐hexane and ethyl acetate fractions in elevated plus maze test.

Group	Open arm		Closed arm	
	Time spent (s)	No. of entries	Time spent (s)	No. of entries
Control (10 mL/kg)	9.60 ± 2.64	1.60 ± 0.25	290.40 ± 2.64	16.80 ± 2.82
Diazepam (1 mg/kg)	107.20 ± 7.56***	10.40 ± 1.99**	192.80 ± 10.80***	6.40 ± 1.57**
MEAC‐200	50.60 ± 4.22***	6.20 ± 0.58***	249.40 ± 4.63***	14.60 ± 1.29
MEAC‐400	80.20 ± 4.02***	9.40 ± 0.93***	219.80 ± 4.02***	10.20 ± 0.86
NHAC‐200	56.80 ± 3.22***	6.0 ± 0.70***	243.20 ± 3.22***	13.20 ± 1.66
NHAC‐400	91.20 ± 4.19***	11.0 ± 1.41***	208.80 ± 4.19***	6.80 ± 1.07*
EAAC‐200	42.80 ± 3.28***	5.60 ± 0.81**	257.20 ± 4.50***	16.60 ± 1.29
EAAC‐200	86.20 ± 4.02***	10.20 ± 1.83**	213.80 ± 3.46***	8.40 ± 1.57*

*Note:* Data were presented as mean ± SEM (*n* = 5), *p < 0.05, **p < 0.01, and ***p < 0.001 considered as significant.

Abbreviations: EAAC, ethyl acetate fractions of MEAC; MEAC, methanolic extract of *A. chama* leaves; NHAC, *n*‐hexane fractions of MEAC.

#### 3.5.2 Hole‐Board Test

**FIGURE 2:**
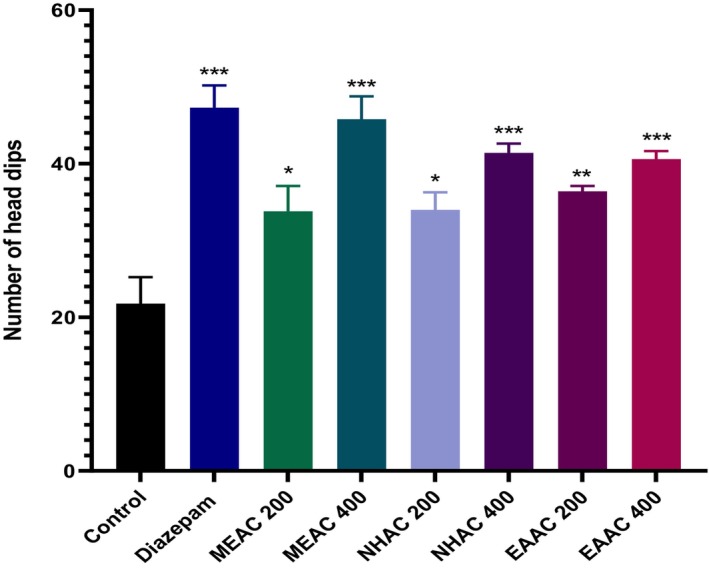
Anxiolytic effects of methanolic extract of *Artocarpus chama* leaves and its n‐hexane and ethyl acetate fractions in the hole‐board test. Data were presented as mean ± SEM (*n* = 5), *p* < 0.01, and ***p < 0.001 considered significant. EAAC, ethyl acetate fractions of MEAC; MEAC, methanolic extract of *A. chama* leaves; NHAC, *n*‐hexane fractions of MEAC.

Significant anxiolytic activity in the HBT was observed at both doses (200 and 400 mg/kg) of MEAC, NHAC, and EAAC, where MEAC led to the greatest number of head dips (45.80 ± 2.99), nearly matching the effect of Diazepam at 1 mg/kg (47.30 ± 2.89) (Figure 2).

### 3.6 In Vivo Sedative Activity

#### 3.6.1 Open Field Test

The OFT results indicated that all treated groups displayed significant sedative effects on Swiss albino mice after 90 min (Table 4). MEAC at 400 mg/kg produced the most pronounced decrease in locomotor activity after 120 min. Diazepam, used as the reference drug, showed marked inhibition of movement beginning at the 60‐min mark and lasting until the conclusion of the test.

**TABLE 4:** Sedative effects of methanolic extract of *Artocarpus chama* leaves and its *n*‐hexane and ethyl acetate fractions in open field test.

Group	Number of Movements (Mean ± SEM)				
	0 min	30 min	60 min	90 min	120 min
Control (10 ml/kg)	88.20 ± 4.02	78.20 ± 4.86	72.80 ± 4.81	69.20 ± 6.55	58.40 ± 2.86
Diazepam (1 mg/kg)	78.80 ± 5.40	46.40 ± 4.61**	26.80 ± 3.84***	18.20 ± 2.54***	14.40 ± 1.75***
MEAC‐200	85.40 ± 3.85	70.20 ± 5.54	48.40 ± 6.02*	40.40 ± 5.49**	36.60 ± 1.44***
MEAC‐400	81.40 ± 4.56	62.80 ± 3.69*	43.80 ± 5.19**	36.20 ± 3.32**	32.60 ± 1.80***
NHAC‐200	82.00 ± 3.91	74.20 ± 5.20	52.40 ± 5.14**	42.60 ± 3.78**	38.80 ± 0.73***
NHAC‐400	82.80 ± 5.03	66.60 ± 2.80	44.00 ± 4.22**	36.60 ± 4.35**	33.00 ± 1.45***
EAAC‐200	87.00 ± 4.35	76.40 ± 3.44	58.60 ± 4.23	47.80 ± 2.76*	41.20 ± 1.32***
EAAC‐400	84.20 ± 2.56	63.40 ± 3.22*	51.80 ± 3.06**	42.20 ± 3.26**	39.40 ± 0.75***

*Note:* Data were presented as mean ± SEM (*n* = 5), *p < 0.05, **p < 0.01, and ***p < 0.001 considered as significant.

Abbreviations: EAAC, ethyl acetate fractions of MEAC; MEAC, methanolic extract of *A. chama* leaves; NHAC, *n*‐hexane fractions of MEAC.

#### 3.6.2 Hole‐Cross Test

In HCT, only treatment groups with 400 mg doses demonstrated notable sedative effects in Swiss albino mice after 90 min. At 120 min, MEAC at a 400 mg/kg dose resulted in the greatest reduction in movement count. Diazepam, the standard drug, exhibited significant suppression of activity starting from 30 min and continued through the duration of the experiment (Table 5).

**TABLE 5:** Sedative effects of methanolic extract of *Artocarpus chama* leaves and its *n*‐hexane and ethyl acetate fractions in hole‐cross test.

Group	Number of movements (Mean ± SEM)				
	0 min	30 min	60 min	90 min	120 min
Control (10 ml/kg)	21.60 ± 1.03	20.60 ± 1.36	19.20 ± 0.86	18.60 ± 0.81	15.40 ± 1.03
Diazepam (1 mg/kg)	20.40 ± 1.21	8.20 ± 0.86***	6.80 ± 1.16***	4.20 ± 0.37***	2.80 ± 0.66***
MEAC‐200	22.00 ± 1.09	19.60 ± 1.21	17.20 ± 0.86	15.80 ± 1.11	14.00 ± 0.71
MEAC‐400	21.00 ± 1.41	16.20 ± 0.97*	13.40 ± 0.51***	12.00 ± 0.84***	8.00 ± 0.44***
NHAC‐200	22.40 ± 1.36	20.20 ± 0.73	16.60 ± 0.92	17.00 ± 1.00	13.00 ± 1.41
NHAC‐400	21.00 ± 1.30	19.80 ± 0.73	13.00 ± 1.14**	15.60 ± 0.75*	10.40 ± 0.75**
EAAC‐200	23.20 ± 1.28	21.40 ± 0.75	18.60 ± 1.08	16.80 ± 0.58	14.00 ± 0.55
EAAC‐400	22.60 ± 1.21	19.60 ± 0.81	18.00 ± 0.95	12.40 ± 0.93**	10.60 ± 0.68**

*Note:* Data were presented as mean ± SEM (*n* = 5), *p < 0.05, **p < 0.01, and ***p < 0.001 considered as significant.

Abbreviations: EAAC, ethyl acetate fractions of MEAC; MEAC, methanolic extract of *A. chama* leaves; NHAC = *n*‐hexane fractions of MEAC.

### 3.7 In Vivo Antidepressant Activity

#### 3.7.1 Forced Swimming Test

**FIGURE 3:**
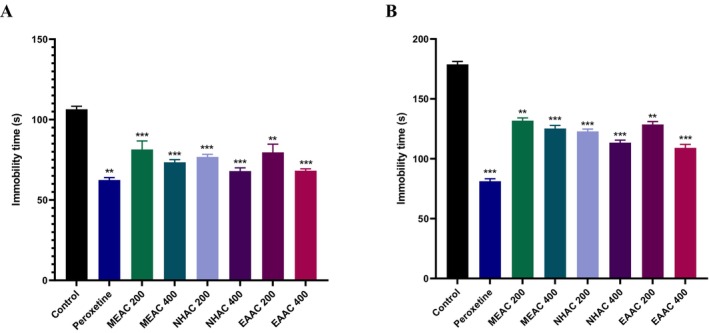
Antidepressant effects of methanolic extract of *Artocarpus chama* leaves and its n‐hexane and ethyl acetate fractions in (A) Forced swimming test and (B) Tail suspension test. Data were presented as mean ± SEM (*n* = 5), **p < 0.01, and ***p < 0.001 considered significant. EAAC, ethyl acetate fractions of MEAC; MEAC, methanolic extract of *A. chama* leaves; NHAC, *n*‐hexane fractions of MEAC.

In FST, notable antidepressant effects were seen at both doses (200 and 400 mg/kg) of MEAC, NHAC, and EAAC. Among them, NHAC exhibited the strongest effect, reflected by the lowest immobility duration of 68 ± 2 s, which is nearly equal to the immobility duration (68.2 ± 1.16) by EAAC (Figure 3A).

#### 3.7.2 Tail Suspension Test

Like FST, significant antidepressant activity in the TST was observed solely at both doses (200 and 400 mg/kg) of MEAC, NHAC, and EAAC, with EAAC demonstrating the highest effects, evidenced by the minimum immobility time of 109.6 ± 2.08 s (Figure 3B).

### 3.8 Molecular Docking Study

Molecular docking is a commonly used computational technique to predict a lead compound's binding affinity toward specific targets (Ali, Noushin, et al. 2024). It is inexpensive, fast, reproducible, and saves considerable time and effort in the drug development process (Ali, Hoque, et al. 2024). The docking results indicated that MEAC‐derived phytoconstituents effectively interacted with drug targets linked to oxidative stress, anxiolytic, sedative, and antidepressant activities. Table 6 summarizes the binding affinity scores obtained from the molecular docking analysis.

**TABLE 6:** Binding affinity of the phytochemicals from methanolic extract of *Artocarpus chama* leaves with the selected target proteins.

Phytochemical	PubChem ID	Antioxidant	Anxiolytic	Sedative	Antidepressant
		1DGH	2Z5X	3U5J	5I6X
1H, 3H‐furo[3,4‐c]furan, tetrahydro—	557908	−4.1	−4.6	−4.3	−4.4
Benzyl alcohol	244	−5.5	−5.6	−5.2	−5.3
1‐butanone, 4‐(dimethylamino)‐1‐phenyl—	547178	−6.4	−6.9	−5.8	−6.4
Octanoic acid, methyl ester	8091	−5.2	−5.4	−4.9	−5.6
Benzylcarbamate	12136	−7.1	−6.8	−6	−6.3
3,5‐methano‐2,3,4,5 (1H)‐tetrahydrobenzo(b)azepin‐2‐one	582678	−7.3	−7.9	−6.9	−7.6
Phenylalanine	6140	−7.4	−7.1	−6	−6.6
2‐methoxy‐4‐vinylphenol	332	−6.1	−6.3	−6	−6.1
Chloroacetic acid, tetradecyl ester	519540	−5.9	−6.5	−5.3	−6.4
N‐hydroxy‐2‐phenyl‐acetamide	220184	−6.8	−6.7	−6.6	−6.2
Fumaric acid, butyl tetrahydrofurfuryl ester	91695626	−6	−7.4	−6	−6.3
4H‐cyclopentacycloocten‐4‐one, decahydro—	558671	−6.6	−7	−6.2	−7
Tetracontane‐1,40‐diol	557624	−5.9	−7.3	−5	−6.5
Tridecanoic acid, 12‐methyl‐, methyl ester	21204	−5.7	−6.9	−5.6	−6.6
6‐hydroxy‐4,4,7a‐trimethyl‐5,6,7,7a‐tetrahydrobenzofuran‐2 (4H)‐one	14334	−6.1	−7.2	−5.9	−7.1
Perhydrophenalene, (3a.alpha., 6a.alpha., 9a.alpha., 9b.beta.)—	123177	−6.5	−7.4	−6.5	−7.4
Neophytadiene	10446	−6.7	−8.1	−6.4	−6.7
Pentyl dotriacontyl ether	91695708	−5.5	−7.4	−5.4	−6.8
Phytyl tetradecanoate	14486554	−6.2	−7.8	−5.2	−7.8
Phytyl palmitate	6437053	−6.2	−8.4	−6.2	−7.4
Trans, trans‐1,6‐dimethylspiro[4.5]decane	565492	−5.8	−6.9	−5.7	−6.7
Tetradecanoic acid, 10,13‐dimethyl‐, methyl ester	554145	−6.2	−7.4	−6.1	−6.9
Tetradecanoic acid	11005	−6.1	−7	−5.7	−6.3
Methyl 11‐methyl‐dodecanoate	4065233	−5.7	−6.9	−5.6	−6.8
2‐hydroxy‐1,8‐naphthyridine	299643	−6.7	−6.8	−6.6	−7
Methyl 10‐trans,12‐cis‐octadecadienoate	5471014	−6.5	−7.7	−5.7	−6.4
Phytol	5280435	−7.2	−8.1	−7.8	−6.9
Heptadecanoic acid, 16‐methyl‐, methyl ester	110444	−6.2	−7.1	−5.8	−6.7
Docosanoic anhydride	566696	−5.5	−6.5	−5.1	−7
Glycidyl palmitate	347736	−6.1	−7.3	−5.4	−6.5
Heptacosanoic acid, 25‐methyl‐, methyl ester	554101	−5.8	−7.8	−5	−6.9
9‐octadecenenamide, (z)—	5283387	−6.7	−7.5	−5.8	−6.8
Hexanedioic acid, bis (2‐ethylhexyl) ester	7641	−6.1	−7.6	−5.5	−6.7
1, 2, 3, 4‐tetrahydro‐3‐(phenylacetamido)quinoline	582648	**−8.7**	**−9.3**	**−8.3**	**−9.4**
Hentriacontane	12410	−5.7	−7.5	−5	−6.3
Bis(2‐ethylhexyl) phthalate	8343	−7.1	−8.2	−6.3	−7.8
Tetrapentacontane	521846	−5	−5	−4.9	−7
Triacontane	12535	−5.5	−7.4	−5.2	−6.4
Octadecanoic acid, 11‐methyl‐, methyl ester	554143	−5.6	−7.5	−5.8	−6.6
Octadecane, 2,6,10,14‐tetramethyl—	521556	−6.5	−8	−5.9	−6.6
Co‐crystalized ligand		−8.3	−8.7	−9.1	−10.8
Standard (ascorbic acid/diazepam/diazepam/fluoxetine)	155903693/3016/ 3016/3386	−5.7	−8.7	−8.9	−9.2

*Note:* Bold letters indicate the best binding score.

#### 3.8.1 Molecular Docking Study for Antioxidant Activity

**FIGURE 4:**
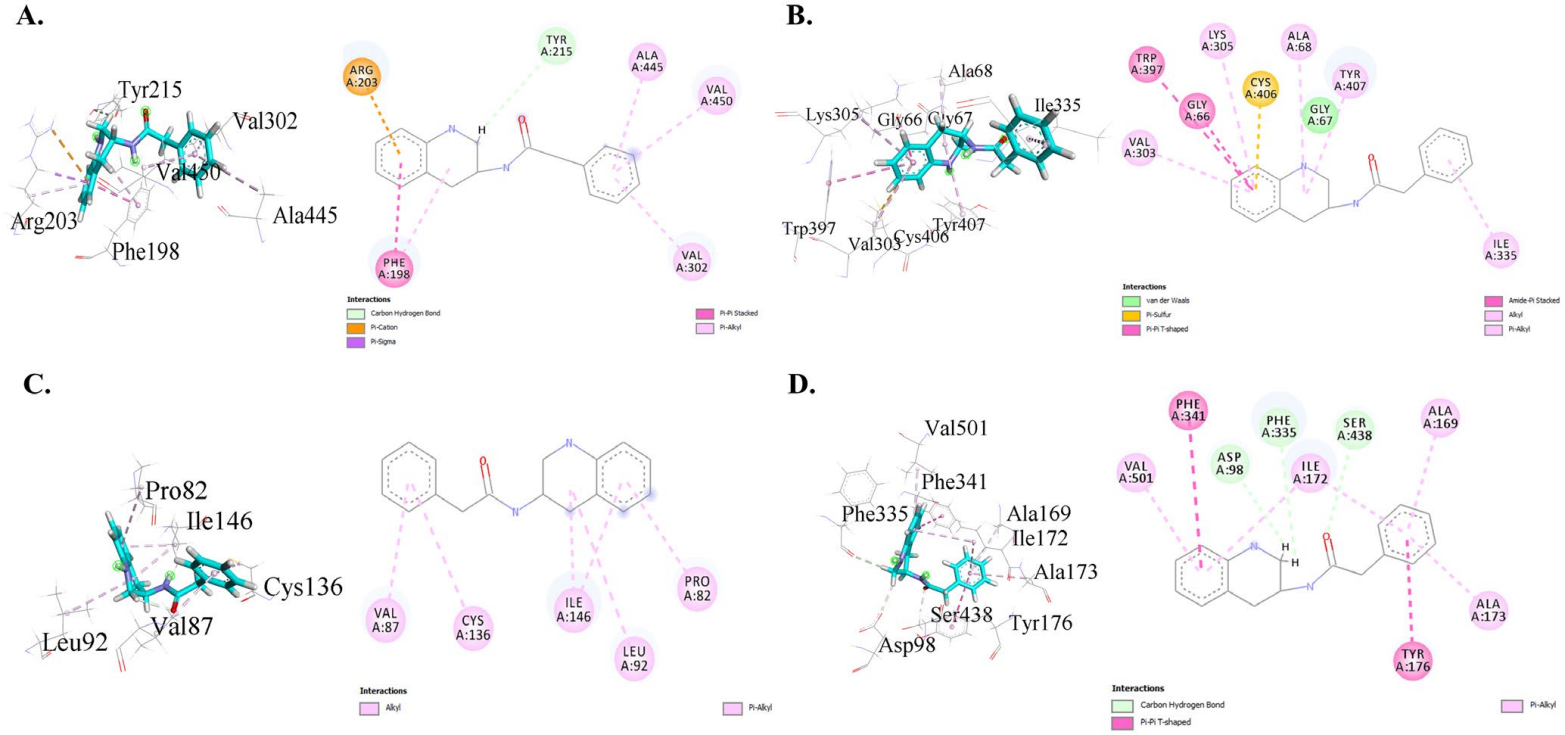
Molecular docking interactions of top docked compound with each selected drug target protein.

Molecular docking results for antioxidant activity are presented in Table 7. 1,2,3,4‐tetrahydro‐3‐(phenylacetamido)quinoline showed the highest binding affinity (−8.7 kcal/mol), followed by phenylalanine (−7.4 kcal/mol); 3,5‐methano‐2,3,4,5 (1H)‐tetrahydrobenzo (b)azepin‐2‐one (−7.3 kcal/mol); phytol (−7.2 kcal/mol); and benzylcarbamate (−7.1 kcal/mol) with human erythrocyte catalase (PDB ID: 1DGH). The co‐crystallized ligand, NADPH, had a score of −8.3 kcal/mol, with 1,2,3,4‐tetrahydro‐3‐(phenylacetamido)quinoline outperforming the co‐crystallized ligand, NADPH. It interacted with the active site, forming bonds with TYR215 and several hydrophobic interactions with PHE198, ARG203, VAL302, ALA445, and VAL450 residues (Figure 4A). Multiple phytochemicals demonstrated higher binding affinities compared to the standard ascorbic acid (−5.7 kcal/mol), aligning with the observed strong in vitro antioxidant activity of *A. chama* leaves.

**TABLE 7:** Docking scores of the top docked compounds identified from methanolic extract of *Artocarpus chama* leaves with the human erythrocyte catalase (PDB ID: 1DGH).

Compound name	Binding affinity	Hydrogen bond interactions		Hydrophobic bond interactions					Electrostatic bond interactions
		Conventional hydrogen bond	Carbon hydrogen bond	Alkyl	Pi‐alkyl	Pi‐sigma	Pi‐Pi stacked	Pi–Pi T shaped	Pi‐cation
1, 2, 3, 4‐tetrahydro‐3‐(phenylacetamido)quinoline	−8.7		TYR215		PHE198 ARG203 VAL302 ALA445 VAL450	ARG203	PHE198		ARG203
Phenylalanine	−7.4	ASN149 GLN195 ARG203	LEU199 ASN149		ARG203		PHE198		ARG203
3,5‐methano‐2,3,4,5 (1H)‐tetrahydrobenzo(b)azepin‐2‐one	−7.3				PHE198 ARG203		PHE198		ARG203
Phytol	−7.2	ASN149	ASN149	ARG203 VAL302 ALA445 VAL450 PRO151	HIS194 PHE198 PHE446				
Benzylcarbamate	−7.1	ASN149 GLN195 ARG203	LEU199 ASN149		ARG203	ARG203	PHE198		ARG203
Co‐crystallized ligand	−8.3	ARG203 ASN213 LYS237 HIS305 SER201 PHE198 TYR215 ASP307	TYR215 ASN149	LYS306					

#### 3.8.2 Molecular Docking Study for Anxiolytic Activity

The results of the molecular docking analysis of phytoconstituents of MEAC with human MAO‐A (PDB ID: 2Z5X), for possible anxiolytic activity, are shown in Table 8. The binding affinities of these compounds ranged from −9.3 to −4.6 kcal/mol. The highest binding affinity was 1,2,3,4‐tetrahydro‐3‐(phenylacetamido)quinoline with a score of −9.3 kcal/mol, whereas for comparison, the co‐crystallized ligand had a lower affinity of −8.7 kcal/mol. Key interactions included pi‐sulfur with CYS406, alkyl with ALA68, and pi‐alkyl with TYR407, VAL303, LYS305, and ILE335 (Figure 4B). The five compounds with the highest binding affinities for MAO‐A were 1,2,3,4‐tetrahydro‐3‐(phenylacetamido) quinoline, phytyl palmitate, bis (2‐ethylhexyl) phthalate, neophytadiene, and phytol. Notably, 1,2,3,4‐tetrahydro‐3‐(phenylacetamido)quinoline, the best‐scoring compound, exceeded the binding affinity of diazepam (−8.7 kcal/mol), indicating its potential role as a lead molecule in the development of new anxiolytics.

**TABLE 8:** Docking scores of the top docked compounds identified from methanolic extract of *Artocarpus chama* leaves with the human MAO‐A (PDB ID: 2Z5X).

Compound name	Binding affinity	Hydrogen bond interactions		Hydrophobic interactions						Other
		Conventional hydrogen bond	Carbon hydrogen bond	Alkyl	Pi‐alkyl	Pi‐sigma	Pi‐Pi stacked	Pi–Pi T shaped	Amide‐pi stacked	Pi‐sulfur
1, 2, 3, 4‐tetrahydro‐3‐(phenylacetamido)quinoline	−9.3			ALA68	TYR407 VAL303 LYS305 ILE335			TRP397	GLY66	CYS406
Phytyl palmitate	−8.4			ARG51 ALA68 VAL210 CYS323 CYS406 ALA448 MET445 ILE180 ILE335 LEU337 VAL93 LEU97	TYR69, PHE208 PHE352 TYR407					
Bis(2‐ethylhexyl) phthalate	−8.2	MET445	TYR444 GLN215	ALA68 ILE180 ILE335 CYS406 ARG51	TYR69 PHE352 TYR407	TYR407				
Neophytadiene	−8.1			LYS305 ILE180 ILE335 LEU337 VAL210	TYR69 PHE208 PHE352 TYR407 TYR444	TYR407				
Phytol	−8.1			ILE180 ILE335 ARG51 CYS406	TYR69 PHE352 TYR407 TYR444	TYR407				
Co‐crystalized ligand	−8.7		GLY67 GLY443	MET445	TYR69 PHE352		TYR407 TYR444			

#### 3.8.3 Molecular Docking Study for Sedative Activity

Table 9 summarizes the results of molecular docking for sedative activity using the bromodomain of human BRD4 (PDB ID: 3U5J). This molecular docking simulation found that 1,2,3,4‐tetrahydro‐3‐(phenylacetamido)quinoline had the highest binding affinity (−8.3 kcal/mol), followed by phytol (−7.8 kcal/mol), 3,5‐methano‐2,3,4,5 (1H)‐tetrahydrobenzo(b)azepin‐2‐one (−6.9 kcal/mol), and n‐hydroxy‐2‐phenyl‐acetamide. The docking score for the co‐crystallized ligand was −9.1 kcal/mol, while the score for 1,2,3,4‐tetrahydro‐3‐(phenylacetamido)quinoline was comparable to the control. Among the docked chemicals, phytol connected with the target protein's active site. It generated six alkyl hydrophobic connections with PRO82, ILE146, VAL87, CYS136, LEU92, LEU94, and MET132, as well as three pi‐alkyl hydrophobic interactions with TRP81, PHE83, and TYR139 residues (Figure 4C). The leading docked compound, 1,2,3,4‐tetrahydro‐3‐(phenylacetamido)quinoline, demonstrated binding affinity closer to the standard diazepam (−8.9 kcal/mol), suggesting its suitability as a prospective lead candidate in drug discovery.

**TABLE 9:** Docking scores of the top docked compounds identified from methanolic extract of *Artocarpus chama* leaves with the bromodomain of human BRD4 (PDB ID: 3U5J).

Compound name	Binding affinity	Hydrogen bond interactions			Hydrophobic bond interactions					Other
		Conventional hydrogen bond	Carbon hydrogen bond	Pi‐Donor hydrogen bond	Alkyl	Pi‐Alkyl	Pi‐Sigma	Pi‐Pi stacked	Pi–Pi T shaped	Pi‐Sulfur
		Amino acid residue	Amino acid residue	Amino acid residue	Amino acid residue	Amino acid residue	Amino acid residue	Amino acid residue	Amino acid residue	Amino acid residue
1, 2, 3, 4‐tetrahydro‐3‐(phenylacetamido)quinoline	−8.3				LEU92 ILE146	PRO82 ILE146 VAL87 CYS136				
Phytol	−7.8				PRO82 ILE146 VAL87 CYS136 LEU92 LEU94 MET132	TRP81 PHE83 TYR139				
3,5‐methano‐2,3,4,5 (1H)‐tetrahydrobenzo(b)azepin‐2‐one	−6.9	ASN140			ILE146 VAL87	ILE146 VAL87 CYS136				
N‐hydroxy‐2‐phenyl‐acetamide	−6.6	ASN135 MET105				ILE146 VAL87 CYS136				
2‐hydroxy‐1,8‐naphthyridine	−6.6	ASN140			ILE146 VAL87	ILE146 VAL87				CYS136
Co‐crystalized ligand	−9.1	ASN140			PRO82 VAL87 ILE146 LEU92	TRP81 PHE83 VAL87 CYS136 ILE146 PRO82	LEU92 ILE146	TPR81	TYR97	

#### 3.8.4 Docking Study for Antidepressant Activity

Docking analysis of antidepressant activity for various compounds is detailed in Table 10, using the human serotonin transporter (PDB ID: 5I6X). Binding affinities ranged from −9.4 to −4.4 kcal/mol, with 1,2,3,4‐tetrahydro‐3‐(phenylacetamido)quinoline showing the strongest affinity at −9.4 kcal/mol. The co‐crystallized ligand had a docking score of −10.8 kcal/mol, and the score of 1,2,3,4‐tetrahydro‐3‐(phenylacetamido)quinoline was close to that of the control. Key interactions included three carbon–hydrogen bonds with SER438, ASP98, and PHE335, four pi‐alkyl interactions with ILE172, VAL501, ALA169, and ILE173, and two pi–pi t‐shaped hydrophobic bonds with TYR176 and PHE341 (Figure 4D). The top five compounds were 1,2,3,4‐tetrahydro‐3‐(phenylacetamido)quinoline, Phytyl tetradecanoate, Bis(2‐ethylhexyl) phthalate, 3,5‐methano‐2,3,4,5 (1H)‐tetrahydrobenzo(b)azepin‐2‐one, and Perhydrophenalene. The best‐docked molecule, 1,2,3,4‐tetrahydro‐3‐(phenylacetamido)quinoline, surpassed standard fluoxetine (−9.2 kcal/mol) in binding affinity, indicating its potential for development as a novel antidepressant agent.

**TABLE 10:** Docking scores of the top docked compounds identified from the methanolic extract of *Artocarpus chama* leaves with the human serotonin transporter (PDB ID: 5I6X).

Compound name	Binding affinity	Hydrogen bond interactions			Hydrophobic bond interactions				
		Conventional hydrogen bond	Carbon hydrogen bond	Pi‐donor hydrogen bond	Alkyl	Pi‐alkyl	Pi‐sigma	Pi–Pi T shaped	Amide‐Pi stacked
		Amino acid residue	Amino acid residue	Amino acid residue	Amino acid residue	Amino acid residue	Amino acid residue	Amino acid residue	Amino acid residue
1, 2, 3, 4‐tetrahydro‐3‐(phenylacetamido)quinoline	−9.4		SER438 ASP98 PHE335			ILE172 VAL501 ALA169 ILE173		TYR176 PHE341	
Phytyl tetradecanoate	−7.8	ARG104	GLN332		ARG104 VAL501 ILE172 LEU99 ILE179 PRO403 LEU406	TRP103 TYR176 PHE334 PHE335 PHE341 PHE407			
Bis(2‐ethylhexyl) phthalate	−7.8	THR497	THR497		VAL501	TYR95 TYR176 PHE335			
3,5‐methano‐2,3,4,5 (1H)‐tetrahydrobenzo(b)azepin‐2‐one	−7.6			PHE341	ILE172	TYR176 ILE172 ALA173		TYR176	SER438 SER439
Perhydrophenalene, (3a.alpha., 6a.alpha., 9a.alpha., 9b.beta.)‐.	−7.4				ILE172	TYR95 PHE341			
Co‐crystalized ligand	−10.8	TYR95 ALA96	ALA169 ALA96 SER336 ASP98 TYR95			TYR95 ILE172 ALA169 ALA173 VAL501		TYR176 PHE341	SER438 SER439

### 3.9 ADMET Investigation

To assess pharmacokinetic and safety parameters, the top three ligands for each protein target from the docking study were evaluated (Table 11). Except for phytyl palmitate and phytyl tetradecanoate, all compounds met the criteria of Lipinski's Ro5, indicating favorable oral bioavailability. Furthermore, no compounds displayed hepatotoxic effects or mutagenicity in the AMES test.

**TABLE 11:** In silico ADMET and drug likeliness study of the selected top docked compounds from the methanolic extract of *Artocarpus chama* leaves.

Compounds name	Absorption		Distribution		Metabolism	Excretion	Toxicity		Lipinski rule of five	Bioavailability
	Water solubility (log mol/L)	Intestinal absorption (Human) (% Absorbed)	VDss (Human) (log L/kg)	BBB permeability (log BB)	CYP3A4 substrate	Total clearance (log ml/min/kg)	AMES toxicity	Hepatoxicity		
1, 2, 3, 4‐tetrahydro‐3‐(phenylacetamido)quinoline	−3.379	93.211	0.3	0.321	Yes	0.273	No	No	Yes	0.55
3,5‐methano‐2,3,4,5 (1H)‐tetrahydrobenzo(b)azepin‐2‐one	−3.108	96.03	0.525	0.118	Yes	0.061	No	No	Yes	0.55
Phytyl palmitate	−5.719	88.577	−0.26	1.035	Yes	1.813	No	No	No	0.17
Perhydrophenalene, (3a.alpha., 6a.alpha., 9a.alpha., 9b.beta.)—	−5.792	96.296	0.592	0.86	Yes	1.102	No	No	Yes	0.55
Phytyl tetradecanoate	−6.328	89.264	−0.137	0.998	Yes	1.776	No	No	No	0.17
Bis(2‐ethylhexyl) phthalate	−6.47	92.45	0.36	−0.175	Yes	1.898	No	No	Yes	0.55
4H‐cyclopentacycloocten‐4‐one, decahydro—	−3.347	95.247	0.36	0.594	No	1.216	No	No	Yes	0.55
2‐hydroxy‐1,8‐naphthyridine	−2.436	92.843	0.059	−0.33	No	0.679	No	No	Yes	0.55

## 4 Discussion

For millennia, medicinal plants have provided therapeutic agents for treating neuropsychiatric disorders, including anxiety, depression, and sleep disturbances (Kenda et al. 2022). Our current investigation focused on assessing the antioxidant potential in vitro and the neuropharmacological effects in vivo of the MEAC and its n‐hexane and ethyl acetate fractions. Furthermore, we employed in silico techniques to examine the likely mechanisms of action and assess the safety of phytochemicals identified via GC–MS analysis.

The genus *Artocarpus*, which has more than 50 species, is widely known for its edible fruits and ethnomedicinal uses. The leaves, bark, and seeds of *Artocarpus* species are reported to have strong pharmacological effects (Jagtap and Bapat 2010). However, only one species, *A. altilis* , has been documented to possess anxiolytic effects. A study by Ajah et al. reported that the methanolic extract of *A. altilis* fruit (breadfruit) demonstrated significant anxiolytic activity in the EPM and LDT models using Swiss albino mice (Ajah et al. 2017). A study by Gonçalves et al. reported that protein fractions of *A. altilis* pulp exhibit antioxidant properties and counteract anxiety behavior in zebrafish. To the best of our knowledge, this is the first study to examine the influence of *A. chama* leaves on neuropsychiatric conditions. Furthermore, it also provides the first evidence of antioxidant activity from the methanolic extract and fractions of *A. chama* leaves (Gonçalves et al. 2020).

Oxidative stress is caused by an imbalance between a high amount of reactive oxygen species (ROS) and inadequate antioxidant defenses. This state can harm cellular components and lead to tissue damage (Preiser 2012). The brain's high lipid composition, constant oxygen demand, and comparatively poor antioxidant defenses make it especially vulnerable to oxidative stress (Bhatt et al. 2020). Anxiety disorders have been associated with reduced antioxidant capacity and increased oxidative damage to proteins, lipids, and nucleic acids. Notably, protein oxidation is proposed as a contributing factor in the development as well as progression of several psychiatric conditions, including anxiety and depression (Fedoce et al. 2018). Our study indicated that the MEAC, along with its n‐hexane and ethyl acetate fractions, exhibited strong antioxidant effects. This antioxidant potential may play a role in their beneficial effects against anxiety and depressive disorders.

EPM, a widely accepted model for evaluating anxiety‐like behavior through fear‐based responses, is commonly employed to screen potential anxiolytic agents (Carobrez and Bertoglio 2005). In our investigation, both doses (200 and 400 mg/kg) of MEAC, NHAC, and EAAC showed significant anxiolytic activity. To further validate these findings, HBT was used, which measures behavioral responses to novel environments and stress, where an increase in head‐dipping frequency indicates reduced anxiety (Olubodun‐Obadun et al. 2023). Consistent with the EPM results, both doses (200 and 400 mg/kg) of each extract showed significant effects in HBT, with MEAC demonstrating the strongest response. One mechanism for alleviating anxiety involves the inhibition of MAO‐A, an enzyme that plays a key role in metabolizing neurotransmitters, endogenous amines, and xenobiotics. Reduced serotonin levels due to increased MAO‐A activity are often associated with anxiety (Libert et al. 2011). In our docking analysis, MEAC phytochemicals showed notable binding affinity to MAO‐A (ranging from −9.3 to −4.6 kcal/mol), suggesting that modulation of MAO‐A could be a significant pathway, among others, through which MEAC exerts its anxiolytic effects. OFT is commonly used to assess exploratory behavior and general physical activity in mice. The key parameter observed is locomotion, where a decline in movement typically reflects sedative properties (Disha et al. 2025). Similarly, HCT also evaluates locomotor activity and serves as a measure of CNS arousal or alertness. Reduced movement in this test suggests lowered CNS excitability and increased calmness or sedation (Hossain et al. 2016). In OFT, treatments with 200 and 400 mg/kg doses of MEAC, NHAC, and EAAC led to a notable reduction in movement across squares, confirming their potential sedative effects. However, only the higher dose (400 mg/kg) of MEAC, NHAC, and EAAC caused significant sedative action in HCT. FST and TST are well‐established behavioral tests used to investigate potential antidepressant effects in mice. These models involve placing the animals in situations of unavoidable stress, prompting a passive coping strategy characterized by immobility (Aziz et al. 2020). This immobile behavior is viewed as a surrogate for human depressive symptoms, particularly feelings of helplessness. Antidepressant drugs are typically found to reduce this immobility, thereby indicating their potential therapeutic efficacy in treating depression (Sofidiya et al. 2022). In both FST and TST, notable antidepressant activity was observed at both of the higher doses (200 and 400 mg/kg) of MEAC, NHAC, and EAAC, evidenced by a significant reduction in immobility time. Among these, EAAC exhibited the most prominent antidepressant‐like effects. A major therapeutic strategy in managing depression involves targeting the serotonin transporter. By inhibiting this reuptake, selective serotonin reuptake inhibitors (SSRIs) increase the amount of serotonin in the synaptic space, thus promoting greater interaction with postsynaptic receptors (Gabrielsen et al. 2012). Molecular docking analysis indicated that the phytochemicals present in MEAC demonstrate strong binding affinity to the active site of the serotonin transporter, suggesting their potential as effective inhibitors.

Neuropsychiatric disorders are frequently linked to imbalances in excitatory and inhibitory neurotransmitters, particularly GABA and serotonin. Consequently, these neurotransmitter systems serve as key therapeutic targets (Islam, Chowdhury, et al. 2025). Diazepam, a benzodiazepine, is one of the most widely prescribed anxiolytics. Benzodiazepines act on a specific binding site of the GABA receptor, producing calming effects, reducing activity, and controlling agitation (Ali, Meem, et al. 2024). Our findings showed that MEAC, NHAC, and EAAC produced anxiolytic responses similar to diazepam in various mouse models, suggesting modulation of the GABAergic pathway (Islam, Hasan, et al. 2025). GABAA receptor activation facilitates chloride influx, which suppresses neuronal excitability and reduces anxiety and sedation (Hasan et al. 2025). Thus, the bioactive constituents of these extracts may potentiate GABAergic signaling, accounting for their observed anxiolytic activity. Moreover, MEAC, NHAC, and EAAC markedly decreased immobility duration in a manner comparable to paroxetine, a recognized SSRI. Since SSRIs are known to reduce immobility in mice, thereby demonstrating antidepressant efficacy, and given the link between immobility responses and hippocampal serotonin transporter activity (Olubodun‐Obadun et al. 2023; Tang et al. 2014), it can be hypothesized that the bioactive compounds of *A. chama* leaves act through modulation of the serotonergic system to mediate their antidepressant effects.

Qualitative phytochemical screening revealed the presence of various classes of compounds, including alkaloids, saponins, phenols, and flavonoids, which may contribute to the biological activities observed in our current investigations. Flavonoids can neutralize ROS by donating hydrogen ions. Moreover, they possess a range of neuropharmacological effects, such as anxiolytic and antidepressant properties, primarily by mitigating oxidative stress and inflammation in the brain (Ko et al. 2020). Additionally, many alkaloids exhibit potent neuropharmacological benefits, largely due to their structural similarity to common neurotransmitters (Kochanowska‐Karamyan and Hamann 2010). GC–MS analysis of MEAC identified forty phytoconstituents from different chemical classes, some of which exhibit central nervous system (CNS) activity and offer neuropharmacological benefits. Benzyl alcohol demonstrated anti‐seizure properties against both focal and generalized seizures (Wu et al. 2019), while phenylalanine, an essential amino acid found in MEAC, has antidepressant effects and may aid in treating depression by enhancing neurotransmitter production through dietary intake (Akram et al. 2020). Phytol, a diterpenoid derived from chlorophyll, exhibited anxiolytic, sedative, and antidepressant effects in mice, along with strong antioxidant activity in the brain (Akram et al. 2020). Neophytadiene, a diterpene, demonstrated anxiolytic and anticonvulsant effects, likely through GABAergic modulation, and also showed mild antidepressant activity (Gonzalez‐Rivera et al. 2023). The presence of these compounds in *A. chama* leaves could contribute significantly to its anxiolytic, sedative, and antidepressant effects.

According to Lipinski's Ro5, which predicts drug‐likeness and oral bioavailability, all eight top‐docked phytochemicals in this investigation conform to the rule, suggesting favorable oral drug‐likeness and potential bioavailability. All selected phytochemicals also demonstrated high intestinal absorption, with values ranging from 88.58% to 96.30%, indicating efficient uptake in the human gastrointestinal system. However, water solubility varied significantly among the compounds. 2‐hydroxy‐1,8‐naphthyridine and the quinoline derivative exhibited better solubility (log mol/L > −3), while lipid‐based compounds such as phytyl palmitate, phytyl tetradecanoate, and bis(2‐ethylhexyl) phthalate were poorly soluble, which may hinder their absorption despite high predicted intestinal permeability. The volume of distribution at steady state (VDss) ranged from −0.26 to 0.592 log L/kg, reflecting variability in tissue distribution. Regarding blood–brain barrier (BBB) permeability, most compounds had log BB values > 0, suggesting the potential to cross the BBB and exert effects on the central nervous system. Notably, phytyl palmitate and phytyl tetradecanoate exhibited particularly high BBB permeability (> 0.99), making them promising candidates for CNS‐targeted therapies. Although most compounds achieved a bioavailability score of 0.55, indicating moderate predicted oral bioavailability, phytyl palmitate and phytyl tetradecanoate scored 0.17, suggesting relatively lower oral bioavailability—possibly due to their poor solubility and lipid‐rich structures. Toxicological evaluation revealed that none of the compounds exhibited AMES toxicity or hepatotoxicity, except for 4H‐cyclopentacycloocten‐4‐one, 1,2,3,4‐tetrahydro‐3‐(phenylacetamido)quinoline, and bis(2‐ethylhexyl) phthalate, which were predicted to be hepatotoxic. These findings emphasize the need for further in vitro and in vivo toxicological assessments, especially for compounds intended for chronic use or CNS applications.

## 5 Conclusion

To conclude, our research highlighted the remarkable antioxidant, anxiolytic, sedative, and antidepressant effects of the MEAC and its n‐hexane and ethyl acetate fractions. Molecular docking studies identified several bioactive compounds with high binding affinity for specific proteins, and the ADMET analysis confirmed their pharmacokinetic and drug‐like properties. Further in‐depth scientific studies are needed to isolate the phytochemicals and investigate their mechanisms of action, as well as to assess their medicinal properties and safety profiles.

## Author Contributions

**Md. Lokman:** conceptualization (lead), data curation (lead), investigation (lead), writing – original draft (equal), writing – review and editing (equal). **Md. Liakot Ali:** formal analysis (equal), software (equal), writing – original draft (equal). **Qurratul Ain Sadia:** formal analysis (equal), software (equal), writing – original draft (equal). **Saad Ahmed Sami:** writing – original draft (equal), writing – review and editing (equal). **Md. Ashraful Alam:** writing – original draft (equal), writing – review and editing (equal). **Nawreen Monir Proma:** project administration (equal), writing – review and editing (equal). **Mohammad Rashedul Islam:** project administration (equal), writing – review and editing (equal). **Mohammed Kamrul Hossain:** resources (lead), supervision (lead), writing – review and editing (lead).

## Ethics Statement

The study was approved by the Animal Ethical Review Board, Faculty of Biological Sciences, University of Chittagong, Bangladesh (approval no. AERB‐FBS‐CU‐20231023).

## Conflicts of Interest

The authors declare no conflicts of interest.

## Data Availability

All relevant data associated with this study will be available upon request to the corresponding author.
